# Acute endometriosis-related sigmoid perforation in pregnancy- case report

**DOI:** 10.1186/s12884-022-04973-9

**Published:** 2022-08-17

**Authors:** S Mittelstadt, A Stäbler, M Kolb, B Krämer, H Horvat, C Reisenauer, C Bachmann

**Affiliations:** 1 Department of Gynaecology and Obstetrics, University Hospital Tuebingen, Calwerstrasse 7, 72076 Tuebingen, Germany; 2 Institute of Pathology and Neuropathology and Comprehensive Cancer Center Tübingen, University Hospital Tübingen, Eberhard-Karls-University, 72076 Tübingen, Germany; 3 Diagnostic and Interventional Radiology, University of Tübingen, Tübingen, Germany

**Keywords:** Deep infiltrating endometriosis, Acute abdomen, Pregnancy, Intestinal perforation, Case report

## Abstract

**Background:**

An acute abdomen is an emergency that requires accurate diagnosis and prompt treatment. In pregnancy, the process is even more challenging and sometimes the radiological findings are unclear. Moreover, endometriosis- related complications are rare, especially in previously unknown endometriosis.

**Case presentation:**

We report on a case of acute endometriosis-related sigmoid perforation during pregnancy (34 weeks of gestation) due to a previously unknown deep intestinal infiltrating endometriosis with focal ulceration of the affected colonic mucosa.

**Conclusions:**

Despite the low relative risk, clinicians should be aware of possible endometriosis-associated complications in pregnancy with potentially life-threatening events, even in previously unknown endometriosis. Further studies should evaluate intestinal complications during pregnancy in relation to previous treatment of intestinal endometriosis (conservative vs. surgical).

## Background

An acute abdomen is an emergency that requires accurate diagnosis and prompt treatment. Delayed diagnosis and treatment can have serious maternal and fetal consequences. Diagnostic tools comprise: ultrasound, CT (computertomography) and MRI (Magnetic Resonance Imaging). The prevalence of endometriosis in women of childbearing age is about 10–12% [1, 2]; intestinal endometriosis is estimated at 5–12% in women affected by endometriosis [1, 2]. It is increasingly reported that pregnant women with endometriosis are at greater risk of adverse obstetric complications [3]. Possible complications are: preeclampsia, preterm birth, (fetus) small for gestational age, antepartum hemorrhage, spontaneous hemoperitoneum, cesarean delivery, stillbirth, postpartum hemorrhage and also spontaneous bowel perforation [3]. To the best of our knowledge the extent of intestinal involvement and the type of prior therapy have not been described so far.

### Aim

We report on a patient with endometriosis-related sigmoid perforation based on a previously unknown deep infiltrating endometriosis (DIE), which also affects the intestinal mucosa and manifests as an acute abdomen in the 34th week of pregnancy, and we have carried out a literature review of the existing literature on this aspect performed.

## Case presentation

A 28-year-old primigravida (spontaneous conception) at 34 weeks' gestation with no relevant comorbidities and an uneventful pregnancy presented to the obstetrics department with a 2-day history of periumbilical pain. The patient had a subfebrile temperature (37,8 °C) and signs of peritonitis (defensive tension). There were no nausea or vomiting. The obstetric ultrasound revealed unremarkable. The infection parameters were increased (leucocytosis:16,000/µL; CRP-levels:11 mg/dL; range:0–0.5 mg/dL). The performed ultrasound and MRI presented no abnormalities and showed no evidence of appendicitis. The started antibiotic therapy led to an improvement in the symptoms, with only discrete pain being detectable and no fever at all. In the following days there was no more fever, the pain was marginal. The re-examination by ultrasound and MRI the following day did not reveal any clear evidence of appendicitis, but sigmoid diverticulitis was suspected due to an enlarged sigmoid wall. The antibiotic treatment was kept. 36 h after starting antibiotic treatment, laboratory results improved and expectant management was maintained. 72 h (3 days later) after presentation, the patient had diarrhea and a flare-up of pain with severe symptoms with acute abdomen, while the laboratory and clinical parameters remained stable (leukocytes 10 160/µg, CRP 6.86 mg/dL and temperature 37.7 °C).

**Fig. 1:**
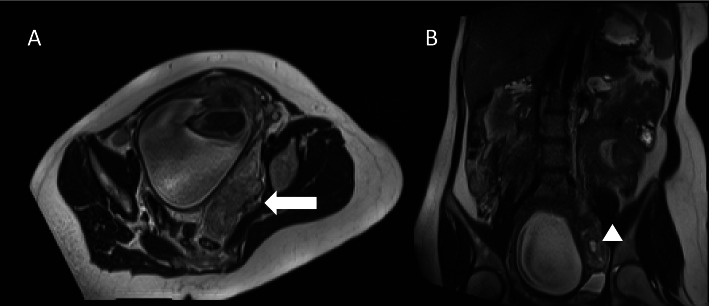
Non contrast-enhanced MRI examination at 1.5 T of the pregnant patient in supine position. **A** transversal T2w HASTE sequence; **B** coronal T2w HASTE sequence. Segmental sigmoid involvement of diverticulitis presenting with diffuse wall thickening (**A** white arrow) and extensive inflammatory changes including a small abscess formation (**B** white arrowhead) and perisigmoid inflammation

**Fig. 2:**
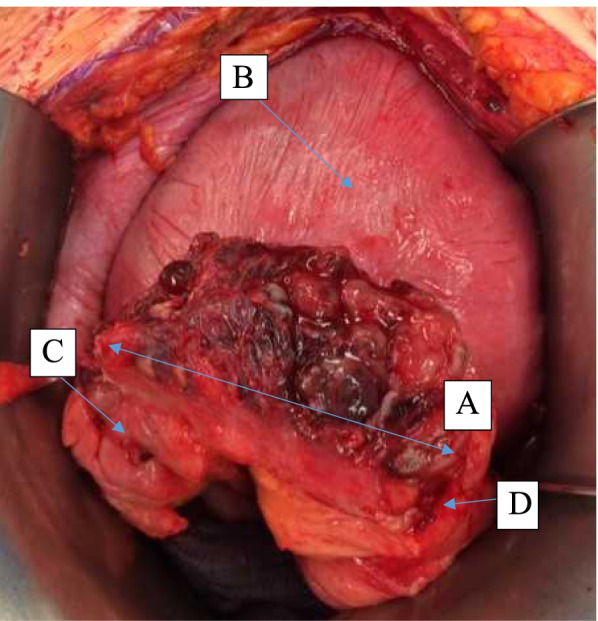
Intraoperative situs with specimen of resected sigmoid colon affected with endometriosis and perforation (**A**) (↑: show distance of specimen); uterus (**B**; ↑); aboral (**C**; ↑) and oral margin (**D**;↑) of sigmoid colon specimen without suspicious tissue

**Fig. 3:**
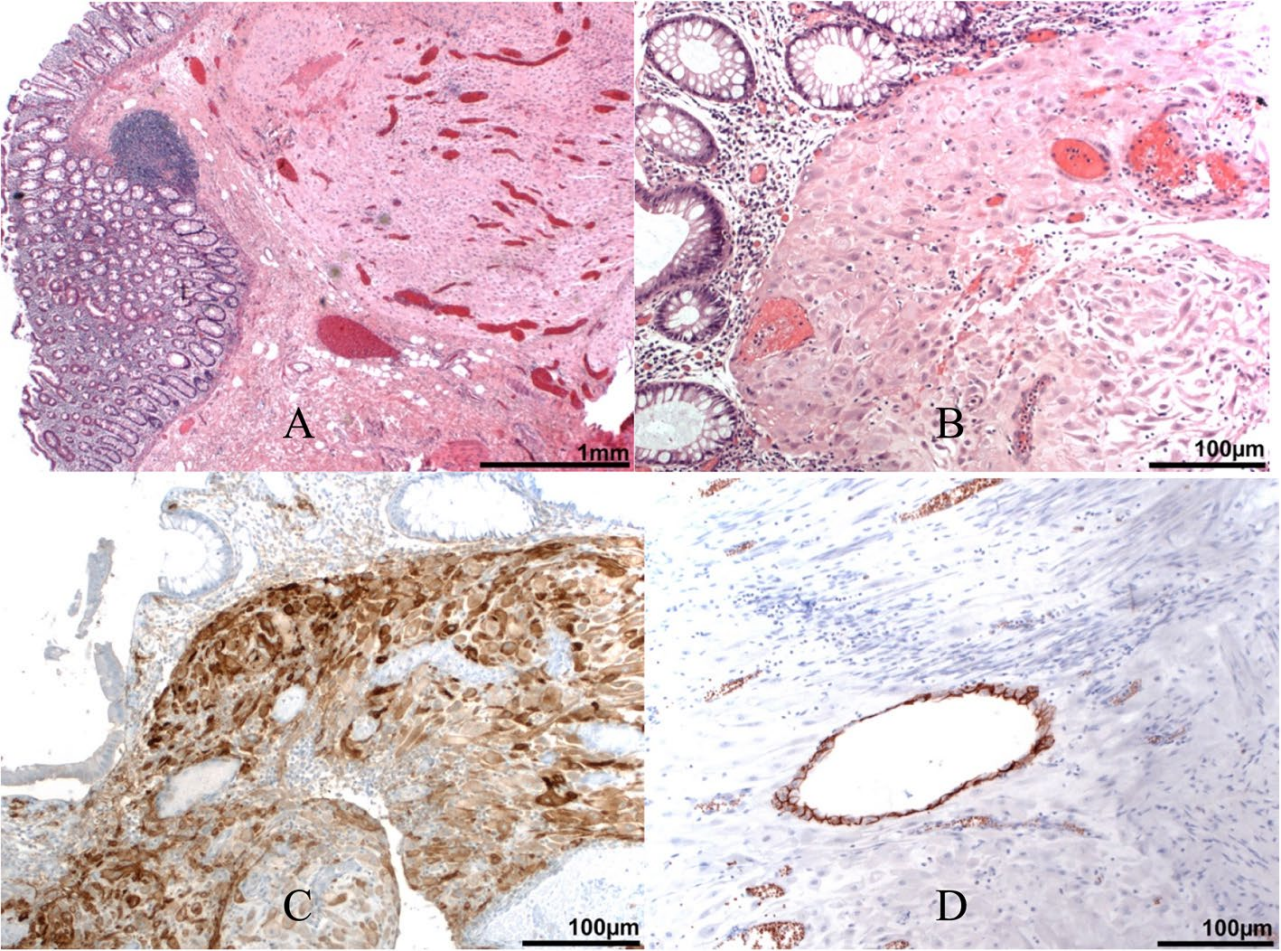
pathology findings: Immunohistochemical analysis was performed on formalin-fixed, paraffin-embedded tissue sections on an automated immunostainer (Ventana Medical Systems, Tucson, AZ, USA). Representative Slides were stained with the following antibodies: BerEP4 (DAKO, Hamburg, Germany), CD10 (Novocastra, Berlin, Germany). Appropriate positive and negative controls were used to confirm the adequacy of the staining. Images were acquired with an Axioskop 2 plus Zeiss microscope equipped with a Jenoptik (Laser Optik System, Jena, Germany) ProgRes C10 plus camera and software. Objectives Plan-Neofluar used were: 1.25/0.035, × 2.5/0.075, × 10/0.30, × 20/0.50 and × 40/0.75. **A** Endometriosis with decidualized endometrioid stroma infiltrating large bowel wall, mucosa and submucosa (25 x). **B** Dezidualized endometrioid stroma in mucosa and submucosa (200x). **C** Immunohistochemistry for CD10 demonstrating endometrioid stroma (200x). **D** Immunohistochmistry for epithelial marker Ber-EP4 demonstrating sparse endometrioid glands in the endometriosis (200x)

The fetal cardiotocography showed also a tachycardia. MRI revealed a sigmoid suspicious for diverticulitis with diffuse wall thickening and extensive inflammatory changes including a small abscess formation (Fig. 1A and B). Therefore, an exploratory laparotomy with C-section was performed. A eutrophic male preterm infant was born at 34 weeks gestation with a good outcome (pHart:7.32; APGAR:9/10/10). The appendix was inconspicuous intraoperatively. A 10 × 10 cm sized conglomerate tumour of the sigmoid colon and left adnexa with abscessing process was detected. A sigmoid perforation with the presence of a tumorous intestinal segment over 5–6 cm was noted. A partial sigmoid resection with side-to-side anastomosis was performed (Fig. 2A-D). There were no other typical or atypical endometriotic lesions in the abdominal cavity. Two red cell concentrates were required to compensate the blood loss of the C-section and diffuse bleeding from the conglomerate tumour. The total blood loss was about 1000 ml. The histological report revealed a large deep endometriotic lesion (4–5 cm) with pronounced abscessing inflammation. The adjacent colonic mucosa was also focally ulcerated (Fig. 3A-D). The patient recovered quickly and had no previous surgeries, denied any symptoms of endometriosis, and had no prior evidence of bowel irregularities.

A progestin-based drug therapy is recommended after surgery with complete resection of DIE to avoid recurrence.

## Discussion

The most common localization of extragenital endometriosis is the gastrointestinal tract [3]. Most affected region is rectum/sigmoid in about 90% [1, 2]. Some patients with intestinal endometriosis are asymptomatic[1]. Intestinal endometriosis is associated with severe, progressive symptoms such as rectal bleeding and rarely, bowel occlusion[2]. Symptoms usually improve during pregnancy, however, some lesions or scars may cause complications [4]. The patient presented had none of these symptoms.

### Therapy of endometriosis

In principle, the surgical approach of DIE depends on the extent of symptoms. The standard of care in symptomatic patients with bowel endometriosis is excision of the lesion or resection of the affected part of bowel; conservative treatment is preferred in asymptomatic patients [5]. DIE requires individual therapy and treatment should be tailored to the patient's symptoms and desires to remove as many lesions as possible while preserving organ function [1]. The surgery should be performed in experienced centers with access to multidisciplinary care [1, 5, 6]. The incidence of bowel perforation is unknown and seems to be rare; especially this aspect had to be considered in patients with or without surgical intervention of intestinal endometriosis.

### Endometriosis-related complications during pregnancy

Endometriosis-related complications during pregnancy have already been described in a few case reports, and possible mechanisms include friability of inflamed tissues and alteration of vessel walls by decidualized lesions [7]. Sometimes superficial endometriotic lesions decidualize during pregnancy and prevent progression [8]. Rarely, nodules of DIE may undergo the process of decidualization during pregnancy [4]. Complications of endometriosis during pregnancy can be mainly attributed to adhesion formation due to the disease itself or previous surgery and chronic inflammation associated with endometriosis, leading to friable tissues [7].

An increased risk of preterm birth, antepartum hemorrhage, delivery from CS, and the rare complications of spontaneous bleeding during pregnancy and spontaneous bowel perforation in patients with endometriosis should be considered [3]. Previous reports mention complications throughout pregnancy [5, 7, 9]. It might be more difficult because of endometriosis related complications (bowel complications) or adverse outcomes such as preterm birth and obstetrical hemorrhages [3, 5, 7]. Complications such as spontaneous bowel perforation, rupture of ovarian cysts, uterine rupture and intra-abdominal bleeding from decidualised endometriotic lesions or previous surgeries have been described previously (Table 1) [9–11]. There is no clear evidence of an increased risk of preeclampsia, having a child born small for gestational age, stillbirth, or postpartum hemorrhage in patients with endometriosis [3, 12]. In this context different risks for complications during pregnancy were demonstrated when comparing spontaneous conception versus assisted reproduction without significant differences in subgroups [9, 13, 14]. Causative factors for complications can be chronic inflammation, adhesions and progesterone resistance [7]. Apart from a premature birth, none of the pregnancy-related complications mentioned above were found in our patient. It is extremely unexpected that the first diagnosis of endometriosis during pregnancy occurs as an acute abdomen in a patient with no history of typical endometriosis symptoms or signs. In particular, the patient presented had deep infiltrating intestinal endometriosis, which also affected the intestinal mucosa. Earlier reports of endometriosis-related bowel perforations describe histological evidence of endometriosis without specifying the extent of intestinal involvement and also without specifying the type of previous endometriosis surgery. Moreover, an endometriosis complication during pregnancy is atypical, as endometriosis improvement is expected [8]. In this context, intestinal perforation during pregnancy is reported very rarely [8, 9]. Intestinal perforations most commonly occur in late pregnancy (26–37 weeks of gestation) or in the postpartum period [8]. The affected region of perforation is most frequently the rectosigmoid, followed by appendix/cecum and small intestine [8]. All reported patients developed an acute abdomen followed by laparotomy [8].

**Table 1:** Summary of cases from the literature review

Author, year	age	Bowel perforation based on endometriosis	Therapy/ surgery	Imaging (ultrasound/MRI) in pregnancy	gestational week	History of endometriosis	symptoms
Petresin J, Wolf J, Emir S, Müller A, Boosz AS**, 2016** [10]	**33y**	**Previously known endometriosis with surgery**	**Laparoscopy adnexectomy ipsilateral**	**Adnexal mass 80 mm**	**15 + **	** + **	**Abdominal pain**
Petresin J, Wolf J, Emir S, Müller A, Boosz AS**, 2016** [10]	**25y**	**Previously known endometriosis (laparoscopy without resection)**			**28 + **	** + **	**Muscular defense, abdominal pain**

### Diagnostic approach of endometriosis during pregnancy

Bowel endometriosis involving the intestinal mucosa, as observed in our patient, is very rare [15]. Its diagnosis is challenging even in asymptomatic patients. In our patient, a perforation was unlikely in the MRI performed, but could not be ruled out due to limited imaging possibilities (Fig. 1A and B). The absence of enlarged lymph nodes or distant metastases favored diverticulitis over sigmoid cancer (Fig. 1A and B). Endometrial tissue implanted in the gastrointestinal tract can cause gastrointestinal symptoms such as abdominal pain, rectal bleeding, and dyschezia [15]. The patient presented had none of these symptoms. We couldn´t identify a specific factor that predicts endometriosis-related complications such as bowel perforation. Several techniques have been proposed including TVS/ transrectal ultrasonography and MRI [1]. These different imaging modalities provide more information about presence, location and extent of endometriosis[1]. The diagnostic performance of TVS and MRI is similar for the detecting DIE affecting the rectosigmoid, uterosacral ligaments and rectovaginal septum [16]. Imaging in pregnant women to carry out an accurate diagnosis is also a challenge, especially in rare pathologies without a pathognomonic sign. Therefore, the foci of endometriosis increase and change their structure due to cellular hypertrophy and stromal edema associated with higher vascularization caused by the hormonal changes in pregnancy [4, 17]. Consequently, these totally benign lesions may resemble malignant tumors in ultrasound examination [4]. TVS is the best diagnostic tool for DIE, especially for rectosigmoid endometriosis [18]. The patient presented had sigmoid endometriosis, which complicated the diagnosis of the disease. It is remarkably difficult to diagnose intestinal endometriosis by conventional imaging methods and remains a challenge [15]; a pregnancy impairs diagnosis as well [15]. Imaging findings can mimic other diseases, including all forms of colitis, acute and chronic inflammatory bowel disease, and many others [15].

### Care in experienced centers

Despite the low relative risk, physicians should pay attention to possible endometriosis-associated complications or complications of previous endometriosis treatment, especially during pregnancy and childbirth [10]. It is important to investigate in a more differentiated manner whether patients have an increased risk of intestinal perforation during pregnancy depending on previous conservative or surgical intervention and their type (discoid excision/ resection). Special clinical attention should be offered to pregnant patients with endometriosis [3]. The rapid decision to operate based on the clinical and radiological findings in the presented case with an atypical surprising diagnosis led to an optimal outcome for mother and child.

In conclusion, the presented case demonstrates that clinicians should be aware of the existence of severe bowel complications during the third trimester of pregnancy in women with DIE [8]. The prevalence of this complication is unknown [8]. If imaging findings are unclear and despite the low relative risk clinicians should be aware of possible endometriosis-associated complications during pregnancy with probable life-threatening events. Further studies are needed to evaluate bowel complications during pregnancy in relation to prior treatment for bowel endometriosis (conservative vs. surgical (excision vs resection)).

## Data Availability

All data generated or analysed during this study are included in this published article. If further information is needed, please feel free to contact the corresponding author.

## Abbreviations

APGAR: Appearance Pulse Grimace Activity Respiration
CT: Computertomography
CRP: C-reactive protein
DIE: Deep infiltrating endometriosis
MRI: Magnetic resonance imaging
pHart: PH arterial
TVS: Transvaginal sonography
